# Gelatin–Sodium Alginate Hydrogels Cross-Linked by Squaric Acid and Dialdehyde Starch as a Potential Bio-Ink

**DOI:** 10.3390/polym16182560

**Published:** 2024-09-10

**Authors:** Joanna Skopinska-Wisniewska, Marta Tuszynska, Łukasz Kaźmierski, Mateusz Bartniak, Anna Bajek

**Affiliations:** 1 Chair of Biomaterials and Cosmetics Chemistry, Faculty of Chemistry, Nicolaus Copernicus University in Torun, Gagarina 7 Street, 87-100 Torun, Poland; 503348@doktorant.umk.pl; 2 Department of Tissue Engineering, Chair of Urology and Andrology, Ludwik Rydygier Collegium Medicum in Bydgoszcz Nicolaus Copernicus University in Torun, Karlowicza 24 Street, 85-092 Bydgoszcz, Poland; lukasz.kazmierski@cm.umk.pl; 3 Faculty of Mechanical Engineering, Institute of Materials Science and Engineering, Lodz University of Technology, Stefanowskiego Str. 1/15, 90-537 Lodz, Poland; mateusz.bartniak@dokt.p.lodz.pl; 4 Department of Oncology, Ludwik Rydygier Collegium Medicum in Bydgoszcz Nicolaus Copernicus University in Torun, Lukasiewicza 1, 85-821 Bydgoszcz, Poland; a.bajek@cm.umk.pl

**Keywords:** gelatin, alginate, hydrogels, squaric acid, dialdehyde starch, 3D printing

## Abstract

Hydrogels as biomaterials possess appropriate physicochemical and mechanical properties that enable the formation of a three-dimensional, stable structure used in tissue engineering and 3D printing. The integrity of the hydrogel composition is due to the presence of covalent or noncovalent cross-linking bonds. Using various cross-linking methods and agents is crucial for adjusting the properties of the hydrogel to specific biomedical applications, e.g., for direct bioprinting. The research subject was mixtures of gel-forming polymers: sodium alginate and gelatin. The polymers were cross-linked ionically with the addition of CaCl_2_ solutions of various concentrations (10%, 5%, 2.5%, and 1%) and covalently using squaric acid (SQ) and dialdehyde starch (DAS). Initially, the polymer mixture’s composition and the hydrogel cross-linking procedure were determined. The obtained materials were characterized by mechanical property tests, swelling degree, FTIR, SEM, thermal analysis, and biological research. It was found that the tensile strength of hydrogels cross-linked with 1% and 2.5% CaCl_2_ solutions was higher than after using a 10% solution (130 kPa and 80 kPa, respectively), and at the same time, the elongation at break increased (to 75%), and the stiffness decreased (Young Modulus is 169 kPa and 104 kPa, respectively). Moreover, lowering the concentration of the CaCl_2_ solution from 10% to 1% reduced the final material’s toxicity. The hydrogels cross-linked with 1% CaCl_2_ showed lower degradation temperatures and higher weight losses than those cross-linked with 2.5% CaCl_2_ and therefore were less thermally stable. Additional cross-linking using SQ and DAS had only a minor effect on the strength of the hydrogels, but especially the use of 1% DAS increased the material’s elasticity. All tested hydrogels possess a 3D porous structure, with pores of irregular shape and heterogenic size, and their swelling degree initially increased sharply to the value of approx. 1000% during the first 6 h, and finally, it stabilized at a level of 1200–1600% after 24 h. The viscosity of 6% gelatin and 2% alginate solutions with and without cross-linking agents was similar, and they were only slightly shear-thinning. It was concluded that a mixture containing 2% sodium alginate and 6% gelatin presented optimal properties after gel formation and lowering the concentration of the CaCl_2_ solution to 1% improved the hydrogel’s biocompatibility and positively influenced the cross-linking efficiency. Moreover, chemical cross-linking by DAS or SQ additionally improved the final hydrogel’s properties and the mixture’s printability. In conclusion, among the tested systems, the cross-linking of 6% gelatin–2% alginate mixtures by 1% DAS addition and 1% CaCl_2_ solution is optimal for tissue engineering applications and potentially suitable for 3D printing.

## 1. Introduction

Hydrogels are three-dimensional structures of cross-linked hydrophilic, synthetic or natural, polymers. The most valuable property of hydrogels is water absorption, which changes as a function of pH, ionic strength, and temperature [1]. Due to their physical state, porosity, and high water content, they imitate natural human tissues much better than typical biomaterials based on synthetic polymers. Biocompatibility, biodegradability, functionality, or reversibility are just a few valuable properties that can distinguish hydrogels. Thanks to these unique properties, hydrogels meet the material and biological requirements for wide biomedical applications. Moreover, the modification of physical and chemical properties of hydrogels is easily possible through, e.g., chemical, physical, and thermal cross-linking reactions [2].

The structure and properties of hydrogels are determined by the presence of specific cross-links between polymer chains that prevent the disintegration of the network. Due to the type of cross-linking bonds, hydrogels can be divided into two groups. First, irreversible gels are cross-linked by stable covalent bonds, where the resulting structure is stiffer and degrades slower. These hydrogels are more stable and, for this reason, they are used as implants and sustained drug release systems. The second group consists of reversible hydrogels cross-linked by weaker bonds, e.g., hydrogen bonds, and hydrophobic or ionic interactions. Such structures are formed quickly and can be rebuilt; they are also usually more responsive to stimuli. This is why reversible gels are often created for biomedical applications [3,4,5]. This extraordinary plasticity and diversity of properties of hydrogels and the ability to create them using various methods make these materials useful also for 3D printing.

Three-dimensional printing is an innovative, rapidly developing technology that enables the production of materials with precisely planned shapes and dimensions. Currently, its use in medical applications is growing dynamically [6]. Over the years, several bioprinting methods have been explored, such as cellular inject printing [7], an extrusion-based method [8,9], and laser-assisted bioprinting [10]. Inject printing has a high printing speed, low cost, and wide accessibility while having the risk of cell exposure to thermal and mechanical stress and inconstant cell encapsulation. Laser-assisted bioprinting stands out as a promising method with good resolutions, but still, the high cost of printing systems and lack of commercial 3D laser bioprinters is a problem. The most common method is the extrusion-based method. Although the extrusion pressure affects cell viability, proper selection of bio-ink and printing parameters may protect them. Precisely controlled 3D structures created with hydrogels determine cells’ morphology and growth characteristics after printing. Significant progress in bioprinting has been demonstrated in the last few years, and as a result, bionic ears [11], artificial bones [12], multi-layered skins [13], and vascular tissues [14] have been created. Its use in medical applications is growing dynamically using various blends of polymers [1]. The use of material with appropriate physicochemical and viscoelastic properties and high biocompatibility, largely provided by hydrogels, is crucial for success of 3D bioprinting strategies.

The simultaneous use of miscellaneous cross-linking methods with different bond formation rates makes hydrogels perfect for 3D printing. It is possible to give them an initial shape, e.g., by quickly forming hydrogen bonds and then further fixing the material’s structure through ionic or covalent cross-linking bonds. Therefore, we decided to use two polymers with different cross-linking mechanisms in our research. These include sodium alginate, which cross-links very quickly in the presence of calcium ions, and gelatin, which forms a hydrogel by forming hydrogen bonds during solution cooling and then can also be additionally stabilized by covalent cross-linking bonds.

Alginate salts are naturally occurring anionic polymers. They are a family of linear, unbranched polysaccharides that contain varying amounts of β-D-mannuronic acid (M) and α-L-guluronic acid (G) residues. The research shows that the presence of α-L-guluronic acid units in the polymer sequence stimulates gel formation, while (1-4)-β-d-mannuronic acid and the mixture of M and G units provide flexibility to the polymer matrix [15]. Combining an aqueous solution of sodium alginate with ionic cross-linkers, such as divalent cations, e.g., Ca^2+^, is the most popular method of preparing gels. Divalent cations are believed to bind only to the guluronic acid fragments in the alginate chain, as these structures provide a high degree of divalent ion coordination. The G blocks of adjacent polymer chains form the so-called egg-box structure in the presence of cations, and as a result, a stable gel is formed. Easily soluble in water, calcium chloride (CaCl_2_) is one of the most widely used agents for the ionic cross-linking of alginate [16]. However, the resulting gels are quite stiff, and obtaining a homogeneous structure with smooth surfaces poses some difficulties. Good biocompatibility and low cost make alginates widely tested as bio-inks. Fast ionic gelation facilitates gel formation but also limits the control of the process during printing. Moreover, the lack of cell adhesion moieties and non-repetitive degradation are strong limitations in their use as bio-ink [11,12,17].

Gelatin is a biopolymer obtained by partial hydrolysis of collagen and exhibits a chemical composition closely similar to the native protein. It comprises characteristic amino acid triplets Gly-X-Y, where Gly is glycine, while X and Y are mainly proline and hydroxyproline. This composition is essential for the gelation properties of gelatin. The polypeptide chain has side functional groups, e.g., –COOH and –NH_2_, which react or interact with acids and bases, aldehydes and sugars, metal ions, electrolytes, and surfactants. As a polar biopolymer, gelatin dissolves quickly in water. Cross-linking of protein chains may affect some physicochemical properties, including mechanical parameters such as tensile strength, stiffness, resistance to enzymatic and chemical degradation, reduction in gas permeation, and cell–matrix interactions [13,14,18,19,20]. Due to its excellent biological properties and support for cell adhesion, gelatin is used as a bio-ink in the production of tissue culture matrices. However, the thermal reversibility of gelatin gels and poor mechanical properties require the use of covalent cross-linking of prints or gelatin modification, e.g., by introducing methacrylic groups enabling photocross-linking of this protein. Therefore, a frequently used method to improve the properties of gelatin materials is to blend the protein with other polymers [12,21,22,23].

Gelatin and alginate are commonly combined in hydrogels for medical purposes. Gelatin provides bioactivity due to the presence of RGD motifs and accelerates the inflammatory response and healing process, while alginate addition improves the mechanical properties of the final material. Moreover, gelatin facilitates the shaping of alginate hydrogels and cross-linking in the appropriate form. Bioprinting is also an area of extensive research using gelatin–alginate hydrogels. The ratio of gelatin to alginate significantly affects the properties of the final hydrogel, which is why many research groups are testing combining these biopolymers in various proportions, from 0.5% to 8% of individual polymers. Often, the predominant component is alginate, whose task is to increase the mechanical strength of the material [24,25]. In other papers, the authors describe systems with a predominance of gelatin providing better conditions for cell adhesion and proliferation [21,23,26,27]. In the case of bioprinting, an additional aspect is the bioprinting parameters, which also strongly affect the material’s properties [28,29,30]. These systems are tested for use in tissue engineering [26,27,30], e.g., bone tissue regeneration [28,29,31], skeletal muscle engineering [32], and meniscal tissue engineering [12]. Gelatin–alginate prints are also considered as drug carriers [33] and dressings for accelerating wound healing [34].

While this polymer mixture is very often used in medical applications and bioprinting, further improvement of the properties of these materials is still possible and desirable. The use of covalent gelatin cross-linking agents may additionally influence the method of gel formation and the properties of the final material [3,32,35,36,37]. Squaric acid (SQ), also known as 3,4-dihydroxy-3-cyclobutene 1,2-dione, is a planar aromatic structure with a unique name due to its unusual square structure. Squaric acid is a highly acidic molecule. The negative charges in the squaric acid dianion are evenly distributed between the oxygen atoms. Therefore, SQ readily reacts with amine groups. SQ is a non-toxic compound and not very aggressive to biomolecules; therefore, it is used in biomedicine. In vitro tests show that SQ is a safer and more effective protein cross-linking agent than previously used compounds, e.g., glutaraldehyde or epichlorohydrin [38,39,40].

Dialdehydes belong to another class of valuable cross-linking agents and include a wide variety of compounds. Due to their unique structural features, dialdehydes can easily engage in cross-link creation. They react with the amino groups of polymers under mild conditions at physiological pH [41]. Hence, some higher molecular dialdehydes, such as poly(ethylene glycol)-dialdehyde (PEG-A) and oxidized polysaccharides, are being tested. Dialdehyde starch (DAS) is an example of modified native starch. It is formed by the oxidation of the hydroxyl groups present in the glucopyranose rings. As a result of the selective oxidation of starch with periodate, the C2-C3 bonds of the starch polysaccharide chain are cleaved, and two aldehyde groups are formed. Dialdehyde starch is biodegradable and non-toxic [42].

The novelty of this research is the use of dialdehyde starch (DAS) and squaric acid (SQ) during multi-stage cross-linking of the gelatin–alginate mixture, which would be formed by the casting method but also allows the creation of structures with a given shape due to 3D printing. We assume that sodium alginate and gelatin will be cross-linked in three ways: in the first stage by forming hydrogen bonds between protein chains, then by ionic interactions between Ca^2+^ and the polysaccharide, and finally by forming covalent bonds between the amino groups of gelatin via squaric acid or dialdehyde starch. As a result, we expect to obtain a biocompatible hydrogel that can be easily formable by 3D printing techniques.

## 2. Materials and Methods

### 2.1. Materials

Gelatin from porcine skin (Type A, ~300 Bloom, suitable for cell culture), sodium alginate (from brown algae, suitable for immobilization of micro-organisms), and 3,4-Dihydroxy-3-cyclobutene-1,2-dione-squaric acid (SQ) were purchased from Sigma Aldrich, Steinheim, Germany, dialdehyde starch (DAS) from CHEMOS GmbH, Altdorf, Germany, and Calcium chloride (CaCl_2_) from Chempur, Piekary Slaskie, Poland.

### 2.2. Hydrogel Preparation

An 18% aqueous gelatin solution and 4% sodium alginate solution in distilled water were prepared. The cross-linkers such as squaric acid (SQ) and dialdehyde starch (DAS) were also dissolved in distilled water. Then, the gelatin and sodium alginate solutions were mixed with the desired amount of various cross-linkers to finally obtain a 6% solution of gelatin and 2% sodium alginate and 1% or 2% of the cross-linker (in relation to gelatin). The mixed solutions were poured onto the bottom of levelled dishes and allowed to gel. After 15 min, all samples were covered with a CaCl_2_ solution and left for 10 min to cross-link. Finally, the samples were washed three times with distilled water to remove excess Ca^2+^ ions.

As a result, a series of materials were obtained as follows: gelatin [%] + sodium alginate [%] + cross-linking agent [%] + CaCl_2_ [%] (Table 1).

### 2.3. Infrared Spectroscopy

Gelatin–alginate hydrogels were air-dried and then cut into thin slices. The FTIR-ATR spectra of samples were recorded using a Thermo Fisher Scientific Nicolet iS10 FTIR spectrophotometer equipped with a Ge single-crystal attachment (Thermo Fisher Scientific, Waltham, MA, USA). Every sample spectrum was recorded in the wavenumber range 4000–600 cm^−1^, with a resolution equal to 4 cm^−1^. Sixty-four scans for each sample were performed. The OMNIC program (Version 9.2.41) was used to analyze obtained results.

### 2.4. Thermal Analysis

The thermal decomposition of air-dried gelatin–alginate hydrogels was measured in the temperature range from 20 °C to 650 °C using the TA Thermoanalizer SDT 650 (TA Instruments, New Castle, DE, USA). The measurement was performed under a nitrogen atmosphere with a gas flow rate of around 50.0 mL/min. The heating rate was 10 K/min. TA Universal Analysis Software (Version 5.5.24) was used to determine the mass loss during material degradation and the temperature values at the maximum speed of the entire process.

### 2.5. Mechanical Properties

The mechanical properties were determined using the Shimadzu EZ testing machine (Shimadzu Corporation, Kioto, Japan). The hydrogel was cut with a die into ten equal strips. Each sample was placed between the metal clamps, carefully gripped, and kept lightly taut. The speed of measurement was 10 mm/min and proceeded until the breaking point of the material. The final results are the mean of at least 5–6 measurements, excluding extreme scores. Young’s Modulus, tensile strength, and elongation at the breaking point were determined. The obtained results were processed using TRAPEZIUMX (Version 1.4.5) software and Microsoft Excel (Office 16).

### 2.6. Swelling Ability

The swelling ability (E_s_) was investigated using the conventional gravimetric method. Air-dried samples of hydrogels were weighed and placed in 0.05 M phosphate-buffer saline (PBS) at pH 7.4, at room temperature. After the appropriate incubation time (1, 2, 4, 6, 24, 48 h), the excess phosphate buffer was removed with absorbent paper, and the material was weighed in a closed vessel. The ratio of weight increase after incubation in PBS solution relative to the initial weight of dry samples was calculated according to the equation E_s_ = ((W_s_ − W_d_)/W_d_) × 100%, where W_s_ and W_d_ are the weights of swollen and dry samples, respectively. The final value is an average of three parallel measurements.

### 2.7. SEM Images

The morphology images of lyophilized gelatin–alginate biomaterials were obtained by a scanning electron microscope manufactured by LEO Electron Microscopy Ltd., Cambridge, UK, 1430 VP model. The sample hydrogels were frozen at −20 °C and lyophilized. Then, a small cubic piece was cut out from the middle of the sample and coated with gold. Visualization of the cross-section of the hydrogel was observed and the pore size was analyzed by the ImageJ program.

### 2.8. Biological Research—Cytotoxicity Test

Biological research was carried out with sterile conditions at all times. The 18% gelatin and 4% sodium alginate solutions were dissolved separately in Dulbecco’s Modified Eagle Medium (DMEM) and then mixed to obtain final concentrations of 6% gelatin and 2% sodium alginate. Cross-linking agents DAS and SQ were prepared in water and PBS, respectively. Then, the gelatin–alginate polymer mixture, DMEM, and cross-linking agents were poured into 50 mL falcons and gently homogenized. Based on the the ISO 10993-5:2009 [43] and 12:2021 norms [44], in each well of the 6-hold plate, gelatin–alginate gels were poured out, to obtain a thin layer (0.5 mm approx.) of the hydrogel. After that, all gels were cross-linked ionically for 10 min by CaCl_2_ solution. Cross-linked gels were cut out into pieces and transferred to the bottom of sterile falcons. Finally, all samples were covered with 3.2 mL of DMEM. The extraction process was carried out for 24 h at 37 °C. After that, the extracts were taken out and added to 3T3 fibroblast cells. A general cytotoxicity assessment by means of an MTT was performed. For statistical analysis, an ordinary one-way ANOVA test and also normality and lognormality tests were performed to determine the statistical significance (*p* < 0.05).

### 2.9. Viscosity Measurements

Polymer solutions (6% gelatin, 2% alginate) with added cross-linking agents (1% DAS, 1% SQ) were prepared according to the methodology described in Section 2.2 and left for 10 min. Then, a viscosity measurement was performed using an Anton Paar MCR 702 Multidrive rheometer (Anton Paar GmbH, Graz, Austria). A measuring set-up consisting of a plate–plate system with diameters of 25 mm was used. The system was thermostated using a Peltier element to a temperature of 37 °C, and then it was kept at the set temperature for 3 min before starting the measurement. The measurement was performed at a constant temperature at a variable shear rate ranging from 0.01 to 1000 1/s.

### 2.10. Printability—Preliminary Test

The 3D printing process was performed using an F-NIS printer provided by SYGNIS S. The ink was prepared according to the methodology presented in Section 2.2. Appropriate volumes of 18% gelatin solution, 6% sodium alginate solution, and DAS or SQ were mixed to obtain a final mixture containing 6% gelatin, 2% sodium alginate, and 1% cross-linker (in relation to gelatin). Before the printing process, the ink was heated up to 37 °C into the cartridge (for 10 min) and then pneumatically extruded onto a print bed with a temperature of 20 °C. Single-layer, fully filled materials were printed through a 0.41 mm diameter nozzle with a print pressure 20–30 kPa. Then, the printed samples were placed in 1% CaCl_2_ solution for 10 min for cross-linking.

## 3. Results

### 3.1. Preliminary Results

In the first stage of the research, the base percentage composition of the gelatin–alginate polymer mixture was established. The mixtures containing 6% gelatin and 1%, 1.5%, 2%, and 3% sodium alginate were ionically cross-linked by 10% CaCl_2_ solution and put into the examination. Initial trials showed unsatisfactory properties of hydrogels containing 1% and 3% sodium alginate. The samples were too weak and too stiff, respectively; therefore, they were excluded from the following research. The results of mechanical tests of hydrogels containing 6% gelatin and 1.5% or 2% sodium alginate are presented in Table 2. The gels containing 2% sodium alginate had a higher strength than other samples; therefore, they were chosen for further study.

**Figure 1:**
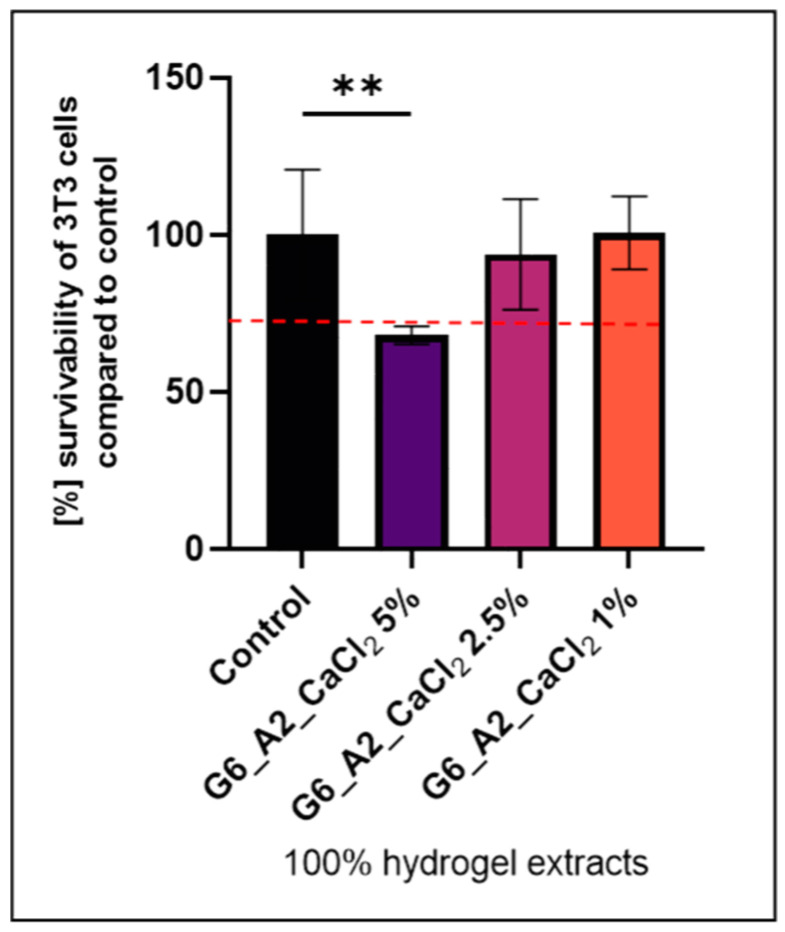
The cytotoxicity test results according to the ISO 10993 norm of hydrogels relative to 3T3 cells (cell survival above 70% is marked with a red intermittent line). ** statistically significant differences.

Additionally, biological research on cell culture survivability by the MTT method, which examines the metabolic activity of cells by creating a coloured product outside of them in DMEM, was carried out. The first cytotoxicity tests showed low vitality of the cells; thus, a high toxicity of tested gels was observed. This was probably caused by the high (10%) concentration of CaCl_2_ solution. Due to the obtained data, it was decided to test samples cross-linked by a CaCl_2_ solution with lower concentrations, that is, 5%, 2.5%, and 1% (Figure 1). Hydrogels formed by cross-linking with 5% CaCl_2_ solution showed a significant increase in relative elongation at break, with a relatively small reduction in the breaking stress, but still had unacceptable cytotoxicity. Superior cell viability, higher than 90%, indicating the material’s biocompatibility, was noticed for gelatin–alginate gels cross-linked using calcium chloride solution in lower concentrations (1% and 2.5%).

The mechanical properties of the hydrogels obtained by cross-linking with a CaCl_2_ solution of reduced concentration were also investigated. The results (Table 2) showed a significant decrease in Young’s Modulus and higher elongation at the breaking point for gels cross-linked by a calcium chloride solution with a concentration lower than 10%. At the same time, the tensile strength of hydrogel cross-linked by 1% and 2.5% CaCl_2_ solution increased. This effect may be due to the more effective diffusion of calcium ions to the inner area of emerging sodium alginate hydrogel when the concentration of CaCl_2_ is lower (2.5% and 1%), and as a result more homogenous structure is formed.

To summarize the preliminary research, the mixture containing 6% gelatin and 2% sodium alginate remained a base mixture for the preparation of all further hydrogels. Two concentrations of the cross-linking solution were chosen. The 2.5% CaCl_2_ solution was chosen due to the more preferable mechanical properties of the hydrogel, and the use of the 1% solution let us obtain the material with the highest biocompatibility.

### 3.2. Infrared Spectroscopy FTIR Analysis

**Figure 2:**
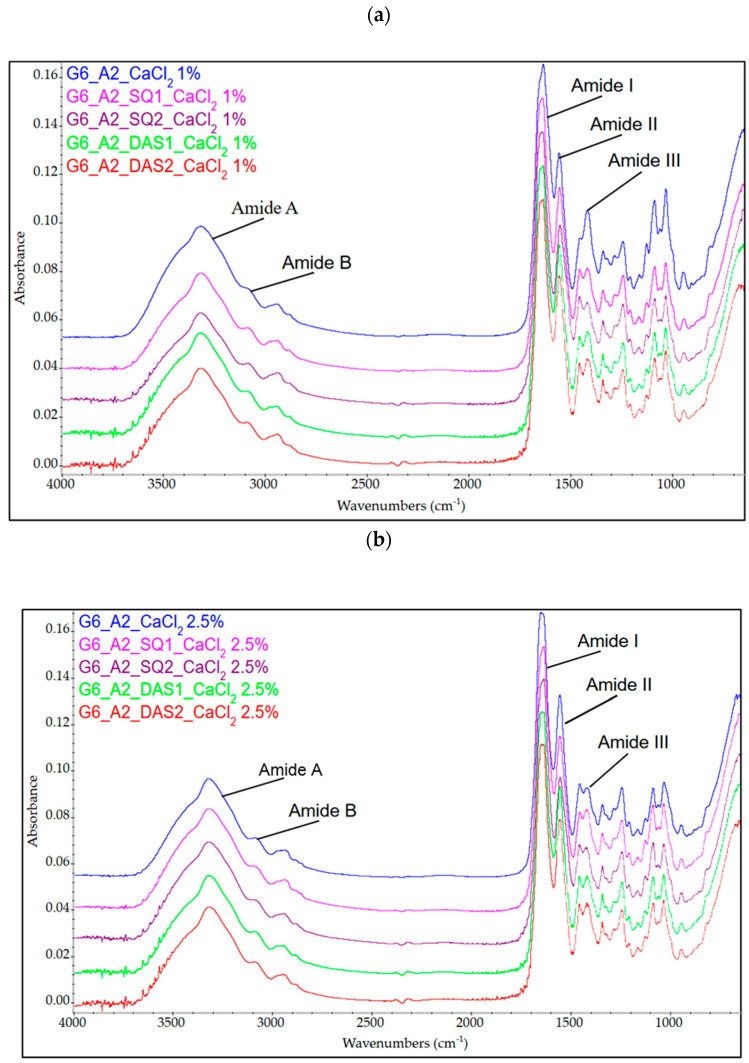
The FTIR spectra of gelatin–alginate materials cross-linked by 1% and 2% SQ and DAS and using (**a**) 1% and (**b**) 2.5% CaCl_2_ solution.

To establish the influence of the cross-linking agents on the molecular structure of gelatin–alginate hydrogels, the FTIR-ATR technique was applied. The FTIR spectra of unmodified and cross-linked gelatin–alginate gels are presented in Figure 2. The bands present on the FTIR spectra of the tested material were assigned to the vibrations of specific groups of atoms (Table 3).

According to the obtained data, the presence of absorption bands characteristic of peptides and polysaccharides was confirmed. One of the most significant bands in the spectrum is the wide band, with the maximum wavelength at 3316–3317 cm^−1^. It arises from O-H and N-H stretching vibrations (Amide A) characteristic of gelatin, which overlaps with stretching vibrations of O-H (at 3358 cm^−1^) typical for alginate. The Amide B band at 3083–3090 cm^−1^ comes from the stretching vibrations of N-H. The peak at 2930–2945 cm^−1^ is characteristic of C-H bonds from both protein and polysaccharide. The Amide I band at 1632–1644 cm^−1^ (CO– and CN– stretching vibrations) and Amide III at 1239–1241 cm^−1^ (C-N stretching bonds, N-H bending bonds) are also observed [13,45]. The band at 1550–1553 cm^−1^ arises from N-H and C-N bond vibrations (Amide II) overlapping with symmetric stretching vibrations of carboxylate salt groups of sodium alginate. Characteristic absorption bands of polysaccharide structure are also present at 1417 cm^−1^ (COO– stretching), 1083 cm^−1^ (C–C and C-O stretching), 1030 cm^−1^ (C–O–C stretching), and 938–945 cm^−1^ (C–O stretching). The stretching vibration band observed at approx. 940 cm^−1^ is specific for the guluronic and mannuronic acids included in the sodium alginate [3,36,46]. The location of the individual band on the gelatin–alginate spectrum is shifted relative to the position of the analogous bands characteristic of pure gelatin [13]. These changes indicate the interaction and good molecular compatibility between gelatin and alginate.

Gelatin–alginate gels cross-linked only by calcium chloride solution did not show significant differences in characteristic band positions. However, the effect of the addition of chemical cross-linkers (SQ or DAS) on the spectrum shape was observed. The FTIR spectrum of gelatin–alginate hydrogels cross-linked by SQ showed slight shifts in specific bands corresponding to amide B, CH_3_, and Amide I and II. Since squaric acid forms covalent bonds with amino groups of proteins and hydrogen bonds with carboxyl groups (Figure 2), the shift to a lower wavelength of amide bands may indicate a partial reconstitution of the polymer network in the tested materials [47]. Moreover, all cross-linker additions also caused a shift to a lower wavelength of the stretching vibration C-O band observed at approx. 940 cm^−1^. This band is attributed to guluronic acid, which is engaged in gel formation due to interactions with calcium ions [48]. The shift in the position of this band suggests that the addition of chemical cross-linkers (SQ and DAS) affects the interaction between alginate and calcium ions [49].

### 3.3. Thermal Analysis Results

**Figure 3:**
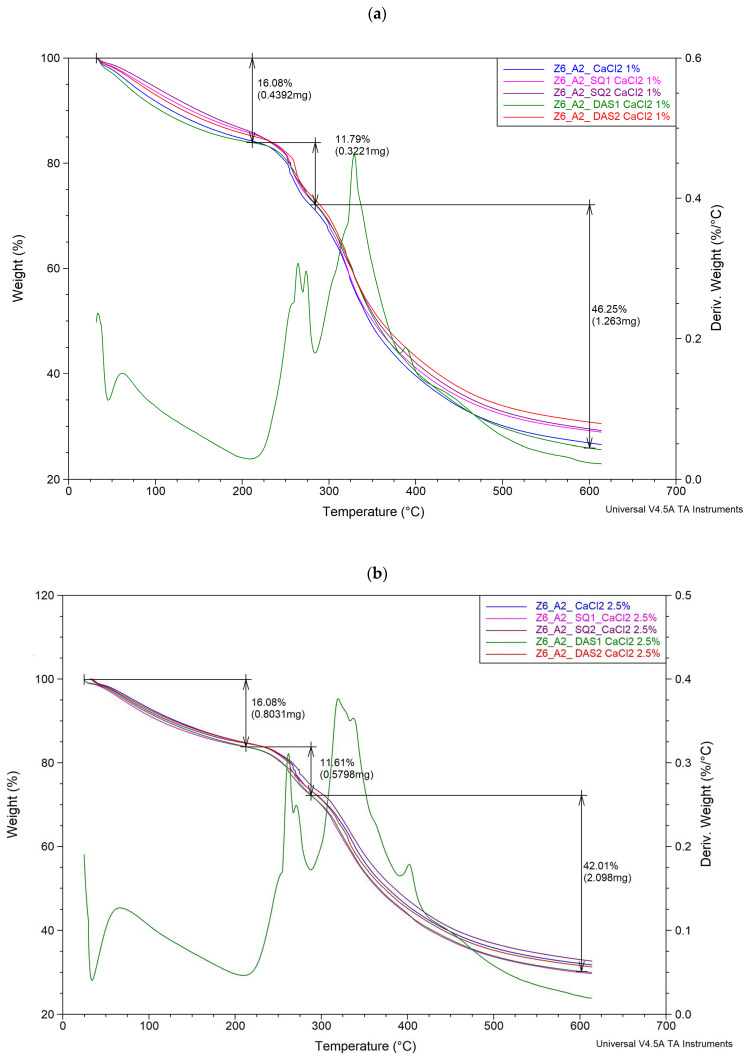
Weight loss (%) of gelatin–alginate gels as a function of temperature, and an example of the first derivative curve of (**a**) G6_A2_DAS1_CaCl_2_ 1% and (**b**) G6_A2_DAS1_CaCl_2_ 2.5%.

The thermal stability of the polymeric material is related to the cross-linking density; therefore, a thermal analysis was performed. The thermogravimetric curves of gelatin–alginate gels cross-linked by Ca^2+^ and SQ or DAS are presented in Figure 3. The parameters of thermal decomposition are presented in Table 4.

The obtained thermograms showed that all materials exhibit similar thermal behaviour. Gelatin–alginate gels presented three thermal degradation steps. The first one occurred at a temperature range of 25 °C to 100 °C, with a weight loss from 14% to 16%. It corresponded to the dehydration of the sample. The second degradation step at a temperature range of 200–300 °C with weight loss of 11% up to 14% was noted and the final stage of decomposition, connected to weight loss from 40% to 45%, occurred at the temperature range 300–600 °C. The second and third steps noticeable in thermograms are a result of many different processes overlapping. We observe here further evaporation of water, but the presence of cross-linking bonds between polymer chains and also the egg-box structures of calcium alginate hinder the diffusion of water molecules through the material and delay water evaporation [50]. At this stage, the melting of gelatin as a physical process takes place, as well as the degradation of the protein chain involving, among others, CO_2_ and NH_3_ molecule release, which indicates the scission of the C-N, C(O)-NH, C(O)-NH_2_, NH_2_, and C(O)-OH bonds of the protein [51,52]. The decomposition of the sodium alginate polysaccharide chain is also indicated. Decarboxylation, breaking C–H bonds, and C–O–C bonds are observed [50,53].

The hydrogels cross-linked with 1% calcium chloride solution showed lower degradation temperatures and higher weight losses than those cross-linked with the 2.5% calcium chloride solution and therefore were less thermally stable. Changes in the thermal parameters of the tested hydrogels were also seen after chemical cross-linking agent addition. However, the observed temperature changes were rather irregular. In most cases, in the second stage of degradation, the addition of the cross-linking agent caused an increase in temperature compared to base samples, except G6_A2_DAS2_CaCl_2_ 2.5%. Meanwhile, in the third stage of thermal decomposition, a slight decrease in the temperature is mostly observed.

### 3.4. Mechanical Properties Test

In order to strengthen the gelatin–alginate hydrogels, and increase their stability and degradation resistance, they were dually cross-linked. Sodium alginate was cross-linked due to interaction with Ca^2+^ and, additionally, gelatin was covalently cross-linked with squaric acid (SQ) and dialdehyde starch (DAS).

**Figure 4:**
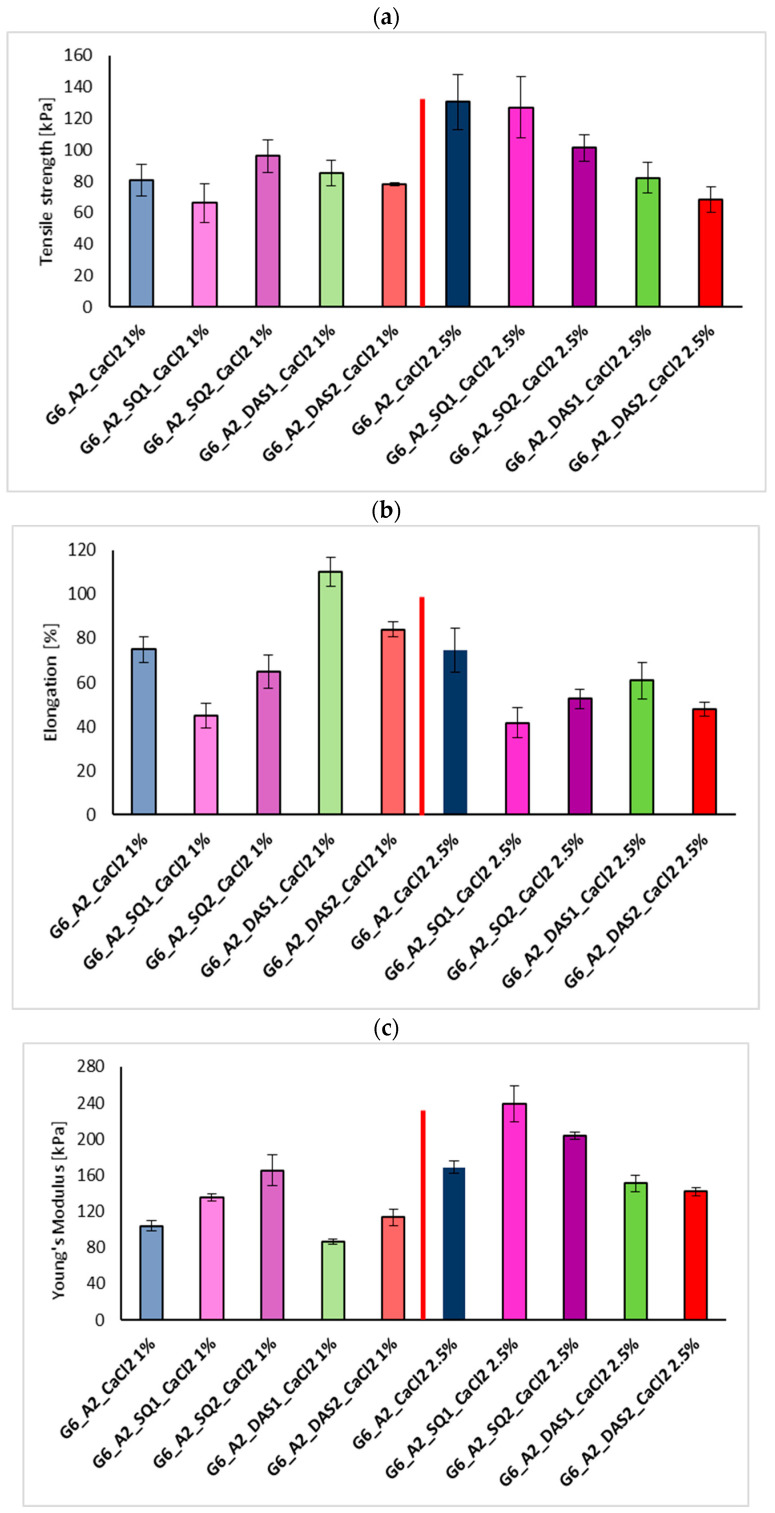
Graphs of mechanical properties of gelatin–alginate gels ionically cross-linked with 1% and 2.5% CaCl_2_ solution and covalently by 1% and 2% addition of SQ and DAS: (**a**) tensile strength [kPa], (**b**) elongation [%], and (**c**) Young’s Modulus [kPa] (vertical red line between the columns is for more clear separation from group cross-linked by 1% CaCl_2_ and group cross-linked by 2.5% CaCl_2_).

Hydrogels’ strength and flexibility rely on their structure, composition, and cross-linking density as well as porosity and water content. The results from the mechanical tests of the gelatin–alginate gels ionically cross-linked with 1% and 2.5% CaCl_2_ solution and covalently by 1% and 2% addition of SQ and DAS are visualized in graphs (Figure 4).

As was previously described, the high concentration of Ca^2+^ ions causes inhomogeneous cross-linking of gelatin–alginate hydrogels, which results in the stiffening of the material but does not improve its strength. Also, the addition of squaric acid and dialdehyde starch affected the mechanical properties of the tested materials. Hydrogels cross-linked by 1% SQ and 1% CaCl_2_ solution presented increased Young’s Modulus values, with a decreased percentage of elongation at the breaking point. The greater the percentage addition of SQ, the higher the observed strength and stiffness of the materials cross-linked with 1% CaCl_2_. Surprisingly, for gelatin–alginate gels cross-linked by SQ and 2.5% CaCl_2_ solution, the opposite trend for mechanical property changes was observed. On the other hand, an unexpected cross-linking effect of DAS on tested materials was seen. The Young’s Module for G6_A2_DAS1_CaCl_2_ 1% significantly decreased with a simultaneous high increase in elongation at the breaking point, indicating better material flexibility and the plasticizing effect of DAS on the tested hydrogels.

### 3.5. Swelling Ability

**Figure 5:**
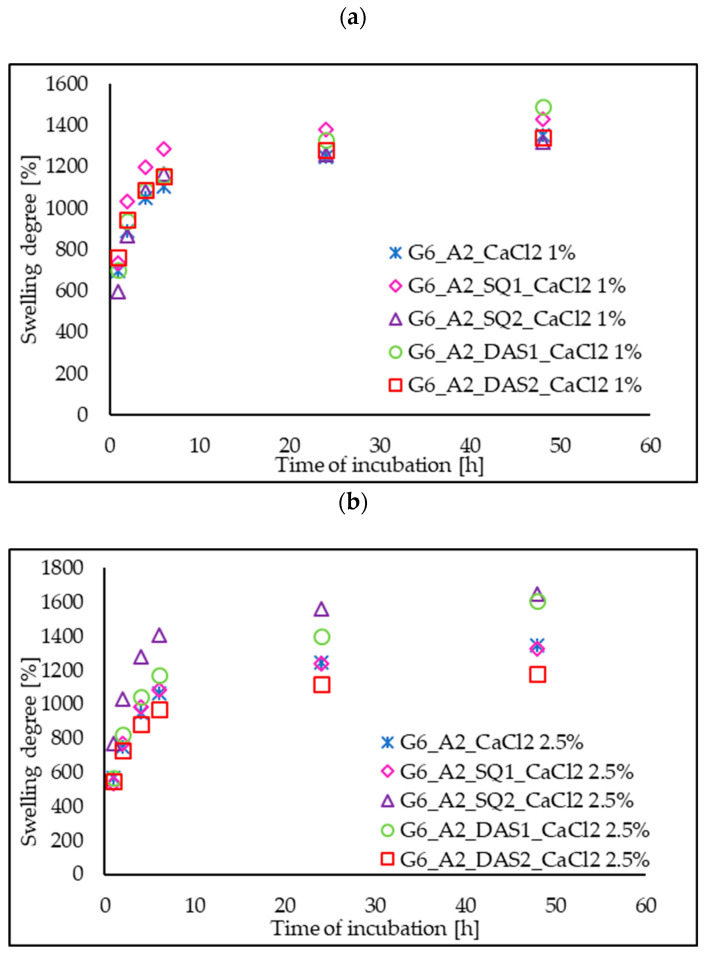
The swelling ratio Es [%] of dry gelatin–alginate hydrogels cross-linked by SQ, DAS: (**a**) 1% and (**b**) 2.5% CaCl_2_ solution.

**Figure 6:**
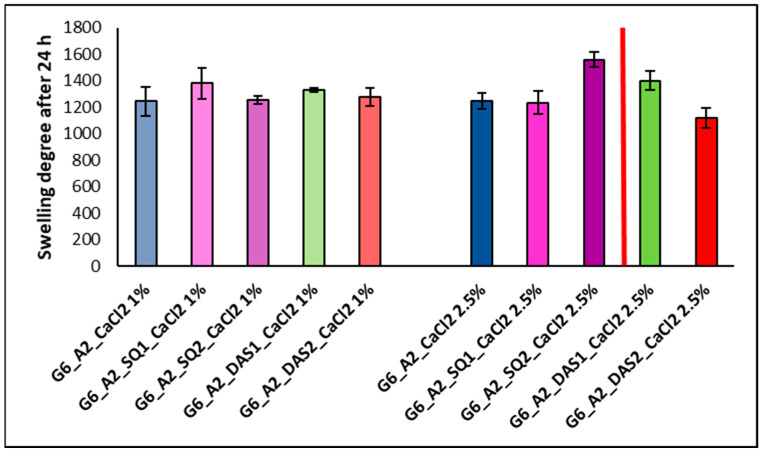
The swelling ratio Es [%] after 24 h of incubation of dry gelatin–alginate gels cross-linked ionically by 1% and 2.5% CaCl_2_ solution and covalently by 1% and 2% SQ and DAS (vertical red line between the columns is for more clear separation from group cross-linked by SQ and DAS).

Water absorption ability is an important feature for tissue engineering scaffolds because it determines the transport of nutrients and waste products. The examined gelatin–alginate gels were exposed to phosphate-buffered saline (PBS) solution in a dry and hydrated state. The effect of cross-linking agent addition (SQ and DAS) and also Ca^2+^ ion concentration on the swelling ratio of the tested hydrogels in PBS was investigated. The swelling degree curves of chemically and ionically cross-linked hydrogels (Figure 5), as well as the comparison of the swelling ratio after 24 h of hydrogel incubation in PBS solution in a dry state (Figure 6), are presented below.

The swelling degree of all the tested gels initially increased sharply to the value of approx. 1000% during the first 6 h, and finally it stabilized at a level of 1200–1600% after 24 h. For comparison, hydrated hydrogels placed in PBS after a 24 h incubation exhibit absorption in the range of 150%. This means that the dried gel returns to its initial hydrated state and behaves similarly to a fresh hydrogel. Base materials G6_A2_CaCl_2_ 1%, and G6_A2_CaCl_2_ 2.5% in a dry state presented a similar swelling degree after 24 h of incubation in PBS. However, the addition of chemical cross-linking agents to gelatin–alginate gels influenced the swelling ability. Even though cross-linking generally reduces the mobility of polymer chains and causes a decrease in swelling ability, we do not observe this effect for gelatin–alginate hydrogels cross-linked by squaric acid and dialdehyde starch. In most cases, the degree of swelling after 24 h of incubation for chemically cross-linked gels is higher than that of base gels, cross-linked only with calcium chloride. This stays in agreement with the results of our previous research on the cross-linking of gelatin hydrogels. Then, it was also observed that the use of highly hydrophilic cross-linking agents increases the material affinity to polar liquids and swelling ability [13].

### 3.6. SEM Observations

**Figure 7:**
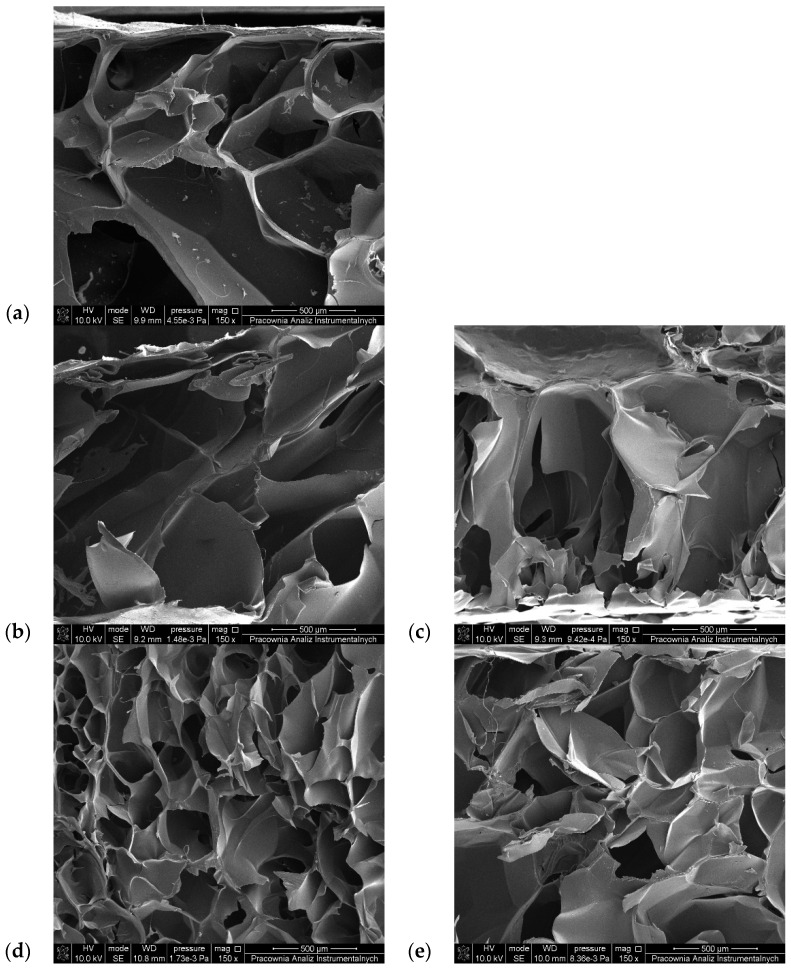
SEM images of lyophilized gelatin–alginate hydrogels: (**a**) G6_A2_CaCl_2_ 1%, (**b**) G6_A2_SQ1_CaCl_2_ 1%, (**c**) G6_A2_SQ2_CaCl_2_ 1%, (**d**) G6_A2_DAS1_CaCl_2_ 1%, and (**e**) G6_A2_DAS2_CaCl_2_ 1%.

**Figure 8:**
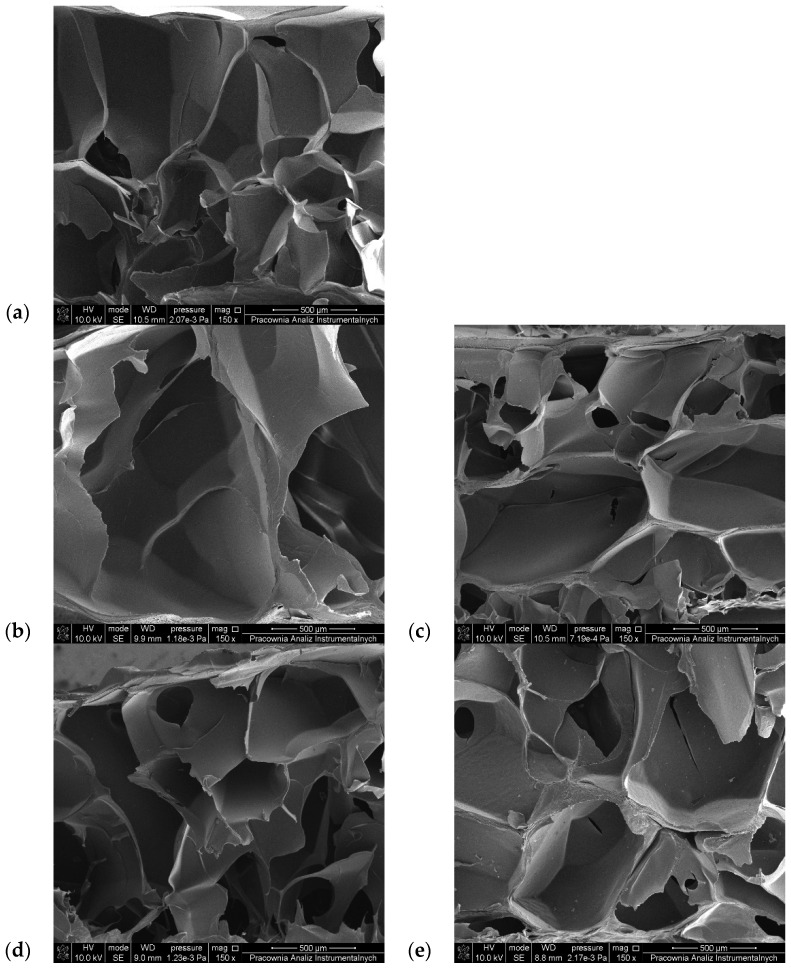
SEM images of lyophilized gelatin–alginate hydrogels: (**a**) G6_A2_CaCl_2_ 2.5%, (**b**) G6_A2_SQ1_CaCl_2_ 2.5%, (**c**) G6_A2_SQ2_CaCl_2_ 2.5%, (**d**) G6_A2_DAS1_CaCl_2_ 2.5%, and (**e**) G6_A2_DAS2_CaCl_2_ 2.5%.

In order to obtain information about the porous structure of the tested materials, SEM images of lyophilized gelatin–alginate hydrogels were prepared (Figure 7 and Figure 8). The pore size of the hydrogels was also estimated and is presented in Table 5.

All the hydrogels possess a 3D porous structure, with pores of irregular shape and heterogenic size. Surprisingly, in most cases, the chemical cross-linking of the hydrogels did not cause tighter packaging of the polymer chains and pore size reduction (Table 5). The average pore size in the gelatin–alginate gels was 347.21 µm for G6_A2_CaCl_2_ 1% and 379.79 µm for G6_A2_CaCl_2_ 2.5%. Only for hydrogels cross-linked with DAS and 1% CaCl_2_ solution was a reduction in pore size observed. In the other materials, the pore sizes are larger despite their covalent cross-linking. This proves that sodium alginate is crucial for forming the three-dimensional structure of the tested hydrogels.

### 3.7. Biological Research—Cytotoxicity Test Results

**Figure 9:**
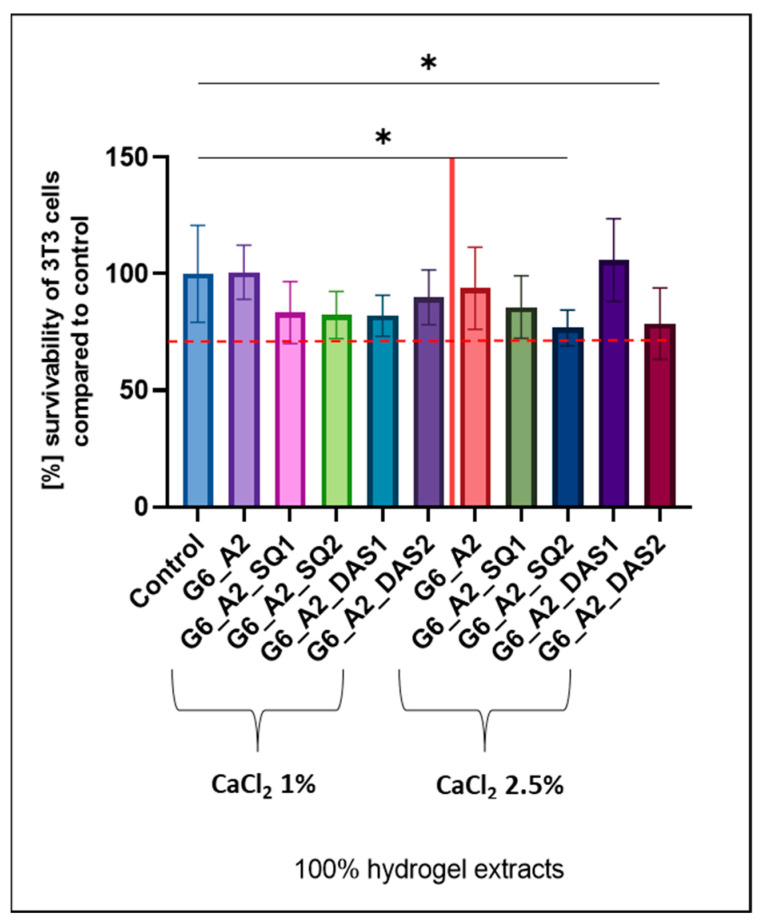
The cytotoxicity extract test results according to the ISO 10993 norm of hydrogels relative to 3T3 cells (cell survival above 70% is marked with a red intermittent line). * statistically significant differences.

A cytotoxicity test is performed to assess the overall toxicity of developed materials. It consists of extracting hydrogels in DMEM according to the ISO 10993-5:2009 and 12:2021 norm and then exposing the cell (3T3) to the extract for a specified time. Evaluation of the cytotoxicity of the tested materials is quantified by the MTT test. This test uses the fact that the dehydrogenase found in living cells reduces the 3-(4,5-dimethyl-2-thiazolyl)-2,5-diphenyl-2H-tetrazoline bromide, known as MTT reagent, to purple formazan. Enzymes from the dehydrogenases group perform the reaction in which the tetrazole ring opens. Thus, the cytotoxicity effect is based on the assumption that only living cells reduce MTT to formazan. The number of living cells by colorimetric measurements is determined [54]. The obtained cytotoxicity results and survivability of cells exposed to 100% of the extract medium form of the gelatin–alginate hydrogels are presented in Figure 9.

The 3T3 cell viability was reduced after 24 h of incubation; however, it still reached more than 70%. 3T3 cells exposed to extracts from the base samples G6_A2_CaCl_2_ 1% and 2.5% revealed high survivability comparable with the control. The addition of chemical cross-linking agents slightly affected the viability of 3T3 cells exposed to extracts from these materials; yet, all significant values remained above the 70% viability established by the ISO 10993-12 standard [55]. Therefore, all the tested gelatin–alginate hydrogels can be considered biocompatible.

### 3.8. Viscosity

**Figure 10:**
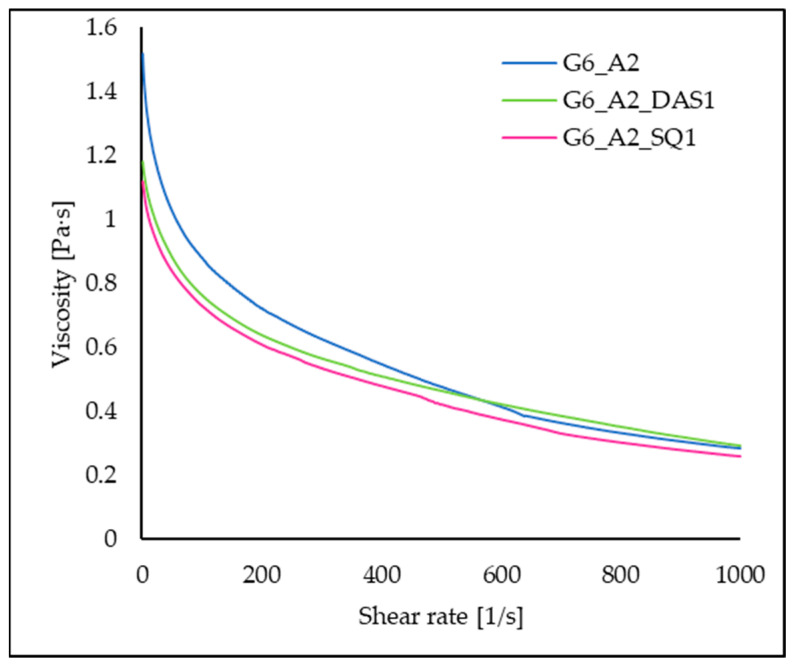
The viscosity of the gelatin alginate solutions with and without the addition of a cross-linker under a shear rate in the range of 0.1 to 1000 s^−1^ at 37 °C.

Rheological properties are crucial for the possibilities of 3D printing. The prepared mixtures of polymers and cross-linking agents were incubated for 10 min, similarly to the ink prepared for printing, to determine the viscosity of the system at the same stage. The curve of viscosity dependence as a function of shear rate for the 6% gelatin–2% alginate solution without and with 1% SQ or DAS addition is presented in Figure 10. It was observed that these solutions are non-Newtonian fluids and are shear thinning. With increasing shear rate, the polymer’s solution viscosity decreases in the range from the initial maximum values of 1.70 Pa·s to 0.28 Pa·s. The addition of SQ reduces the viscosity of the polymer solution in the entire range of shear rates (from 1.16 Pa·s to 0.26 Pa·s), while the viscosity of G6_A2_DAS1 is initially lower, and above a shear rate of 600 1/s it becomes slightly higher than the viscosity of the solution without cross-linking additives (from 1.24 Pa·s to 0.29 Pa·s). Shear thinning is a desirable feature of bio-inks. It allows the reduction in the pressure used during printing and the associated shear stresses, which makes it possible to limit cell damage during extrusion [21]. The above results also indicate that the formation of covalent bonds between gelatin chains via DAS or SQ is slow. During the first 10 min, we observe the dilution of solutions, and the cross-linking degree affecting the properties of the material is achieved only after a longer time, which is confirmed by the results of other studies, e.g., mechanical properties and thermal stability.

### 3.9. Printability—Preliminary Test

**Figure 11:**
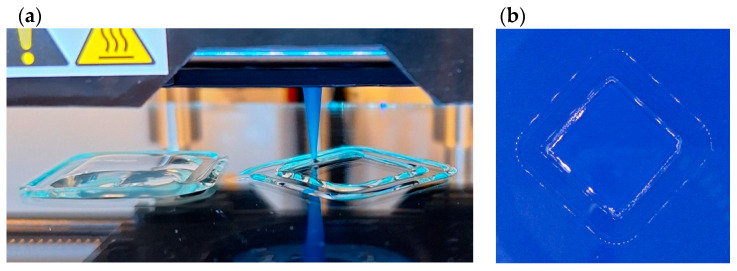
Three-dimensional printing process (**a**) and printout (**b**) of G6_A2_DAS1.

Due to the planned combination of the bio-ink with cells, it was assumed that the temperature of the material in the cartridge should be as close to 37 °C as possible, which is optimal for cell culture. Therefore, the printing process was tested at temperatures of 35 °C, 37 °C, and 40 °C. No difficulties were observed in printing the assumed shape at any of the temperatures, so it was decided that the optimal bio-ink temperature was 37 °C. The chemical cross-linking of gelatin with DAS occurred slowly and did not affect the printability at least 30 min after the preparation of the components (at this time, the prints were finalized). Cooling the printing plate to 20 °C enabled the rapid stabilization of the hydrogel shape through thermal gelling of gelatin. This allowed the print to be safely placed in the cross-linking solution, and ionic bonds between sodium alginate and calcium ions to be created. As a result, uniform prints with dimensions of 20 mm × 20 mm and equal thickness throughout the entire area were obtained (Figure 11). We believe this preliminary test is a good starting point for optimizing the gelatin–alginate hydrogel printing method.

## 4. Discussion

Gelatin is a polymer that is characterized by the thermal mechanism of gelation. When the temperature goes down, hydrogen bonds are formed between the protein chains and stabilize the structure. However, non-cross-linked gelatin gels are thermally reversible. Due to this sensitivity to temperature changes, gelatin can be easily formed in different shapes, for instance, during layer-by-layer 3D printing with simultaneous cooling [24]. However, the mechanical properties and durability of gelatin hydrogels are often insufficient. Alginates show quick gelation in the presence of divalent ions, creating stable, relatively stiff gels, but they are generally inert in relation to cells. So, the combination of gelatin–alginate allows the rapid preparation of quite stable hydrogels and promotes cell attachment and proliferation [3,35,36,56].

On the basis of preliminary studies, it was found that 6% sodium alginate and 2% gelatin is the optimal concentration of polymer solution for the preparation of gelatin–alginate hydrogels and suitable for 3D printing. It was observed that the concentration of the calcium chloride solution used for cross-linking has a significant impact on the properties of the hydrogel. It is well known that alginates form hydrogels in the presence of divalent cations. This process is very fast and leads to the creation of a well-organized “egg-box” structure. Initially, a solution of calcium chloride with a concentration of 10% was used for hydrogel cross-linking. However, the materials prepared in this way were found cytotoxic. Calcium ions play an important role in the cultivation of eukaryotic cells and are involved in the regulation of many life processes. However, its concentration in the cytoplasm must be low, because calcium ions in high concentrations damage the cell membrane, which leads to an abnormal level of cell electrolytes. For this reason, too many calcium ions in the environment may cause cytotoxicity [16,57,58]. Moreover, we observed the rapid formation of the highly cross-linked outer layer, which prevents the cross-linking agents from reaching the deeper areas of the material. Consequently, the inner part of the hydrogel is unproperly cross-linked and less stable. This resulted in the stiffening of the material but did not improve its strength effectively. The reduction in the calcium chloride solution concentration to 1% or 2.5% CaCl_2_ slowed down the cross-linking process, and the curst layer preventing the diffusion of ions inside the hydrogel was not formed on the surface, and a more homogenous structure of the hydrogel was created [59]. As a result, the hydrogels prepared in this way were more elastic but still relatively strong and, just as importantly, biocompatible. Therefore, the hydrogels containing 6% gelatin, 2% sodium alginate, and cross-linked using 1% or 2.5% CaCl_2_ solution were chosen for the next experiments.

**Figure 12:**
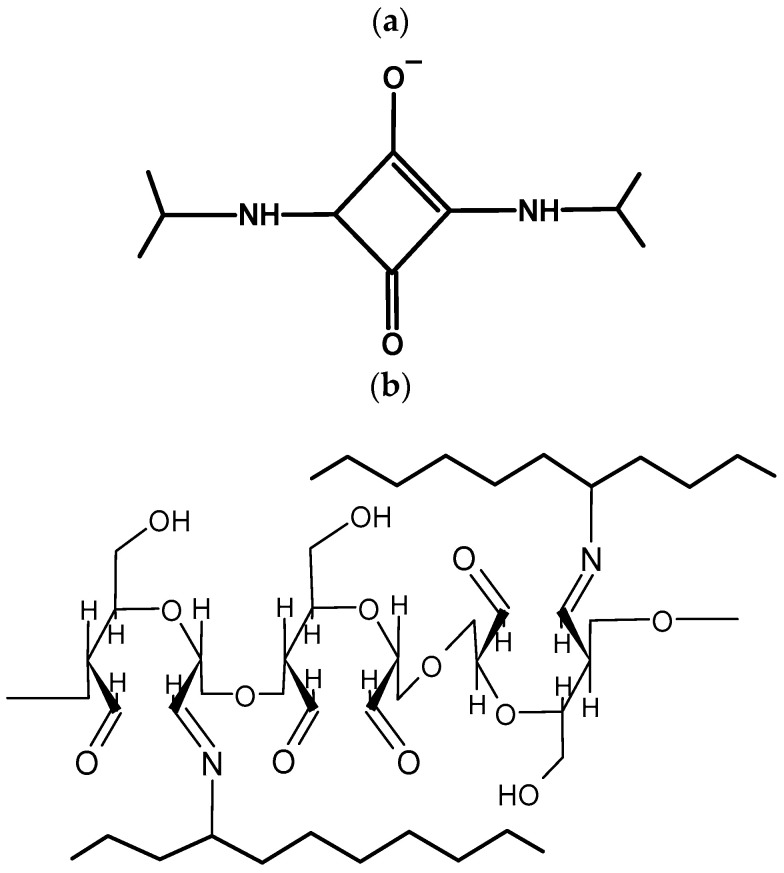
The cross-linking bond shame between amino groups of a protein and (**a**) squaric or (**b**) dialdehyde starch.

To further improve the mechanical properties and durability of the gelatin–alginate hydrogels, they were also cross-linked using chemical cross-linking agents: squaric acid (SQ) and dialdehyde starch (DAS). Squaric acid, as well as dialdehyde starch, forms covalent bonds with amino groups of proteins and hydrogen bonds with carboxyl groups (Figure 12). However, this process is much slower than the formation of ionic interactions. As a result, the viscosity of the tested solutions initially decreases slightly after adding DAS or SQ. We assume that this process is slow enough in our system to allow the extrusion of the bio-ink.

The increase in the temperatures of degradation stages proves the occurrence of the cross-linking process and forming a more thermally stable structure. However, irregular changes in degradation temperatures and weight loss may also indicate that the addition of chemical cross-linking agents affects the creation of bonds between sodium alginate and calcium ions [45,60]. Also, the shift of amide band positions to a lower wavelength proves a partial reconstitution of the polymer network in the tested materials [47]. Moreover, all cross-linker addition also caused the shift to a lower wavelength of the stretching vibration band observed at approx. 940 cm^−1^. This band is attributed to guluronic acid, which is engaged in gel formation due to interactions with calcium ions [48]. The shift in the position of this band proves that the addition of chemical cross-linkers (SQ and DAS) affects the interaction between alginate and calcium ions [49]. This strongly suggests the presence of competitive interactions of calcium ions and cross-linking agents with the polymers.

The obtained data show that the mechanical properties of the materials depend on the combined effect of several factors, such as the stabilizing effect of Ca^2+^ ions on sodium alginate and the interaction of disparate chemical cross-linkers with gelatin [61]. However, we have to bear in mind that the addition of chemical cross-linkers also affects the interactions between the calcium ions and the sodium alginate as well as the organization of the alginate structure. Our previous studies have shown that the pure gelatin gels cross-linked by DAS become stiffer [13]. But the current experiments give different results. The Young Modulus for the gelatin–alginate hydrogels dually cross-linked with CaCl_2_ solution and dialdehyde starch decreases because the addition of DAS disrupts the formation of the egg-box structure.

Moreover, gelatin has cationic and anionic regions, while sodium alginate is an anionic polymer. It causes strong electrostatic interactions between the polymer chains and affects the emerging three-dimensional structure of the hydrogel [62,63]. This attraction and repulsion of different fragments of the macromolecules may be the main reason for the unusual porous structure formation. The pore architecture plays a vital role in tissue regeneration by providing temporary mechanical function, swelling ability, preserving tissue volume, and circulating nutrients and waste products [36]. So, despite the cross-linking of the hydrogel structures, only a slight decrease, and in some cases, even higher swelling capacity, is observed. We must also take into account that both chemical cross-linking agents (SQ and DAS) are hydrophilic compounds that can affect the increased water absorption capacity of the material [64,65,66]. On the other hand, the additional ionic cross-linking reduces swelling ability. These two opposite trends significantly complicate the phenomenon of gradually swelling cross-linked gelatin–alginate hydrogels. As one can see, many factors affect the swelling ability of the obtained hydrogels; however, what is most important, all the tested materials exhibit PBS absorption in the range of 1100–1500% from a dry state, which is acceptable from the point of view of using these materials in tissue engineering.

To sum up, various interactions occur between the components of the hydrogel: electrostatic interactions between polymer chains, ionic bonds between calcium ions and carboxyl groups of alginate, and covalent bonds involving the amino groups of gelatin formed by cross-linking agents. All these interactions directly affect the structure of the hydrogel, and thus its mechanical properties, stability, porosity, and swelling capacity. The highly hydrated network structure enables the exchange of gases and nutrients, which is an attractive option for creating inks for bioprinting. The presented gelatin–alginate hydrogels find promising applications in intensively developing 3D bioprinting due to presented physicochemical characteristics such as appropriate biological and chemical properties, and cross-linking speed.

## 5. Conclusions

This research concerns the development of biopolymer hydrogels suitable for tissue engineering. The potential possibility of using the mixture of gelatin–alginate and cross-linking agents in 3D printing was also assessed. During this study, the properties of gelatin–alginate hydrogels cross-linked ionically by CaCl_2_ solution in concentrations of 1% and 2.5% and covalently by 1% and 2% squaric acid and dialdehyde starch were examined. We found that high calcium ion concentrations adversely affect 3T3 cell survival, and a reduction in CaCl_2_ solution concentration used for alginate cross-linking is preferred. Moreover, the multi-step cross-linking of gelatin–alginate hydrogels significantly influences their properties. The stabilizing effect of Ca^2+^ ions on sodium alginate and the interaction of chemical cross-linkers (SQ and DAS) with gelatin led to the formation of hydrogels with a modified structure. The presence of gelatin, and especially cross-linked gelatin, hinders the formation of alginate “egg-box” structures in the presence of calcium ions. This complex combination of various processes causes the hydrogels to be more homogeneous, more equally cross-linked, and, at the same time, flexible. However, the properties of hydrogels with different compositions obtained by various cross-linking technics are difficult to predict theoretically and each system requires experimental testing to determine its properties. Moreover, due to the complicated mixing procedure and specific interaction of squaric acid with sodium alginate, dialdehyde starch is an easier-to-use cross-linking agent for gelatin–alginate materials. Taking into account the above results, it was concluded that the material containing 6% gelatin and 2% alginate cross-linked with 1% dialdehyde starch addition and 1% CaCl_2_ solution (G6_A2_DAS1_CaCl_2_ 1%) is the most promising for application in tissue engineering. Our experiments confirmed the possibility of using the developed material in 3D printing. In the following research stage, we will include cells in the developed ink and study the possibility of directly creating cell-laden constructs.

## Figures and Tables

**Table 1:** Examples of names and shortcuts of hydrogels used in this work.

Sample Full Name	Shortcut
Gelatin 6% + Sodium Alginate 1.5% + Calcium Chloride 10%	G6_A1.5_CaCl_2_ 10%
Gelatin 6% + Sodium Alginate 2% + SQ 1% + Calcium Chloride 5%	G6_A2_SQ1_CaCl_2_ 5%
Gelatin 6% + Sodium Alginate 2% + DAS 2% + Calcium Chloride 2.5%	G6_A2_DAS2_CaCl_2_ 2.5%
Gelatin 6% + Sodium Alginate 2% + Calcium Chloride 1%	G6_A2_CaCl_2_ 1%

**Table 2:** Strength parameters of gelatin–alginate gels ionically cross-liked by CaCl_2_ solution with various concentrations.

Sample	Tensile Strength [kPa]	Elongation at the Breaking Point [%]	Young’s Modulus [kPa]
G6_A1.5_CaCl_2_ 10%	53.79 ± 8.03	40.71 ± 3.78	139.40 ± 12.76
G6_A2_CaCl2 10%	61.49 ± 5.12	28.42 ± 3.82	274.80 ± 23.98
G6_A2_CaCl_2_ 5%	56.17 ± 11.52	91.98 ± 10.33	199.12 ± 11.05
G6_A2_CaCl_2_ 2.5%	130.44 ± 17.62	74.68 ± 9.95	168.95 ± 7.02
G6_A2_CaCl_2_ 1%	80.64 ± 10.02	74.99 ± 5.78	103.93 ± 5.99

**Table 3:** The position of the main bands [cm^−1^] in the FTIR spectra of gelatin–alginate hydrogels cross-linked ionically by 1% and 2.5% CaCl_2_ solution and covalently by 1% and 2% SQ and DAS.

Sample	Amide A	Amide B	CH_3_	Amide I	Amide II	Amide III	C=O Sym.	C-O C-C	C-C C-O-C	C-O
G6_A2_CaCl_2_ 1%	3316	3090	2940	1632	1553	1240	1416	1084	1030	945
G6_A2_SQ1_CaCl_2_ 1%	3316	3089	2940	1635	1551	1240	1416	1084	1030	941
G6_A2_SQ2_CaCl_2_ 1%	3317	3083	2934	1636	1550	1239	1417	1084	1030	938
G6_A2_DAS1_CaCl_2_ 1%	3316	3090	2942	1633	1552	1240	1417	1083	1030	938
G6_A2_DAS2_CaCl_2_ 1%	3316	3090	2941	1633	1552	1240	1417	1084	1030	938
G6_A2_CaCl_2_ 2.5%	3316	3090	2940	1632	1552	1240	1417	1083	1029	942
G6_A2_SQ1_CaCl_2_ 2.5%	3317	3090	2935	1633	1552	1240	1417	1084	1030	941
G6_A2_SQ2_CaCl_2_ 2.5%	3317	3090	2941	1633	1551	1241	1417	1083	1030	941
G6_A2_DAS1_CaCl_2_ 2.5%	3317	3089	2941	1644	1552	1240	1417	1083	1030	938
G6_A2_DAS2_CaCl_2_ 2.5%	3317	3089	2940	1644	1552	1240	1417	1084	1030	938
Gelatin	3308	3080	2940	1644	1551	1239	1454	1082	1032	973
Sodium alginate	3358	-	2934	1600	-	1124	1412	1087	1029	948

**Table 4:** The parameters of the thermal decomposition of gelatin–alginate hydrogels unmodified and cross-linked using SQ, DAS, and 1% and 2.5% CaCl_2_ solution.

Sample	I Stage	II Stage		III Stage	
	Δm [%]	T [°C]	Δm [%]	T [°C]	Δm [%]
G6_A2_CaCl_2_ 1%	15.77	256	12.09	323	45.31
G6_A2_SQ1_CaCl_2_ 1%	14.40	256	12.99	320	43.45
G6_A2_SQ2_CaCl_2_ 1%	14.09	255	13.57	330	42.79
G6_A2_DAS1_CaCl_2_ 1%	16.08	264	11.79	329	46.25
G6_A2_DAS2_CaCl_2_ 1%	14.83	261	11.52	320	42.86
G6_A2_CaCl_2_ 2.5%	15.22	268	11.94	336	40.74
G6_A2_SQ1_CaCl_2_ 2.5%	16.11	271	12.20	323	41.69
G6_A2_SQ2_CaCl_2_ 2.5%	15.33	274	11.59	333	40.04
G6_A2_DAS1_CaCl_2_ 2.5%	16.08	274	11.61	229	42.01
G6_A2_DAS2_CaCl_2_ 2.5%	15.20	264	11.31	332	41.88

**Table 5:** The pore size of gelatin–alginate hydrogels covalently cross-linked using SQ 1% and 2% and DAS 1% and 2% and ionically cross-linked by 1% and 2.5% CaCl_2_ solution.

Sample	Pore Size (µm)	Sample	Pore Size (µm)
G6_A2_CaCl_2_ 1%	347.21 ± 49.71	G6_A2_CaCl_2_ 2.5%	379.79 ± 38.66
G6_A2_SQ1_CaCl_2_ 1%	428.05 ± 102.28	G6_A2_SQ1_CaCl_2_ 2.5%	504.31 ± 218.23
G6_A2_SQ2_CaCl_2_ 1%	477.44 ± 91.20	G6_A2_SQ2_CaCl_2_ 2.5%	445.31 ± 120.81
G6_A2_DAS1_CaCl_2_ 1%	235.94 ± 42.76	G6_A2_DAS1_CaCl_2_ 2.5%	425.03 ± 58.43
G6_A2_DAS2_CaCl_2_ 1%	330.41 ± 22.68	G6_A2_DAS2_CaCl_2_ 2.5%	561.16 ± 109.29

## Data Availability

Data are contained within the article.
